# Measurement of Cardiac Index by Transpulmonary Thermodilution Using an Implanted Central Venous Access Port: A Prospective Study in Patients Scheduled for Oncologic High-Risk Surgery

**DOI:** 10.1371/journal.pone.0104369

**Published:** 2014-08-19

**Authors:** Stéphanie Suria, Anne Wyniecki, Alexandre Eghiaian, Xavier Monnet, Grégoire Weil

**Affiliations:** 1 Service d'Anesthésie, Gustave Roussy, Villejuif, France; 2 Service de réanimation médicale, Hôpital de Bicêtre, Le Kremlin-Bicêtre, France; 3 Unité de recherche EA4533, Université Paris-Sud, Le Kremlin-Bicêtre, France; Central South University, China

## Abstract

**Background:**

Transpulmonary thermodilution allows the measurement of cardiac index for high risk surgical patients. Oncologic patients often have a central venous access (port-a-catheter) for chronic treatment. The validity of the measurement by a port-a-catheter of the absolute cardiac index and the detection of changes in cardiac index induced by fluid challenge are unknown.

**Methods:**

We conducted a monocentric prospective study. 27 patients were enrolled. 250 ml colloid volume expansions for fluid challenge were performed during ovarian cytoreductive surgery. The volume expansion-induced changes in cardiac index measured by transpulmonary thermodilution by a central venous access (CIcvc) and by a port-a-catheter (CIport) were recorded.

**Results:**

23 patients were analyzed with 123 pairs of measurements. Using a Bland and Altman for repeated measurements, the bias (lower and upper limits of agreement) between CIport and CIcvc was 0.14 (−0.59 to 0.88) L/min/m^2^. The percentage error was 22%. The concordance between the changes in CIport and CIcvc observed during volume expansion was 92% with an r = 0.7 (with exclusion zone). No complications (included sepsis) were observed during the follow up period.

**Conclusions:**

The transpulmonary thermodilution by a port-a-catheter is reliable for absolute values estimation of cardiac index and for measurement of the variation after fluid challenge.

**Trial Registration:**

clinicaltrials.gov NCT02063009

## Introduction

Major surgery is associated with a high level of morbidity and mortality. Oncologic surgery often consists in major surgery such as cytoreductive surgery for advanced ovarian cancer, cytoreductive surgery or hyperthermic intraperitoneal chemotherapy for peritoneal carcinomatosis. Ovarian cancer cytoreductive surgery includes total abdominal hysterectomy, bilateral salpingo-oophorectomy, omentectomy, appendicectomy, peritonectomies, pelvic and para-aortic lymphadenectomies. Bowel resections, splenectomy, caudal pancreatectomy, large stripping of the peritoneum removing more than 5 cm^2^ and liver resection may be necessary [1]. Hemodynamic monitoring coupled with adapted therapy reduces postoperative mortality and morbidity for moderate and high-risk surgical patients [2].

In our institute, we apply a local protocol for cardiovascular management associating hemodynamic monitoring and intervention with fluid challenge and/or additional inotropic support. For very high-risk surgery, we chose to monitor the cardiac index (CI) with the transpulmonary thermodilution technique (TPTD), as previously described [3]. The technique is based on the injection of a cooled bolus of saline into a central vein via a central venous catheter (CVC) [4]. Nowadays, we usually place a femoral arterial catheter and a jugular CVC before surgery. The CVC is inserted on the opposite side of the permanent implantable venous port. Indeed, most of the patients treated in our hospital are fitted with a port for chemotherapy or parenteral nutrition. Insertion of the CVC can be more difficult because of the port [5]. But no evidence was found about the possibility of using port for transpulmonary thermodilution.

The aim of this study was to assess whether measurement of the CI by TPTD was possible and reliable via the port. We conducted a prospective study comparing the measurement of the CI by TPTD before and after fluid challenge via the port versus the CVC.

## Materials and Methods

### Ethics statement

The study was approved by the regional Ethics Committee (CPP Ile de France VII, SC12-006, January 2012). As we did not conduct a therapeutic study, Ethics Committee approved the use of an oral informed consent. It was preoperatively given by patients after written and oral information. After oral consent, every patient was recorded in the database of medical research department of our institute. A data book of the protocol with a description and the list of the demographic, clinic and biologic data necessary to be manually collected during the study were placed in every anesthetic file. The trial was registered on ClinicalTrials.gov with registry number 02063009.

We conducted prospective study between March and June 2012 at Gustave Roussy Institute.

All consecutive patients fitted with a port, undergoing major gynecologic surgery requiring measurement of CI by TPTD by the PICCO2 device (Pulsion Medical Systems, Munich, Germany) in accordance with our local hemodynamic protocol were enrolled. This technique allows a correct hemodynamic monitoring even if the diaphragm is open or an epidural analgesia is used after the surgery (specific conditions with limits of the pulse contour analysis).

Patients younger than 18 years old were excluded. Exclusion criteria included contraindicated use of a CVC or failure to insert the CVC. We also excluded patients in whom the use of a port was contraindicated: local infection, general infection and no blood backflow.

Anesthesia cares were given by one of the investigators and an anesthesia nurse. All patients were monitored using pulse oximetry, capnography, non-invasive arterial pressure, the bispectral index (Covidien, Elancourt France), EKG, neuromuscular transmission and temperature monitoring. After the induction of general anesthesia, a femoral arterial catheter ((Pulsiocath PV2015L20N, Pulsion Medical Systems, Munich, Germany) was placed and an ultra-sound guided CVC (Multi-Lumen Central Venous Catheter CV 12703, 7 Fr, 16 cm, Arrow, Teleflex Medical) was inserted contra laterally to the port. Correct position of the tip of the catheter (superior extremity of the right atrium) was controlled with fluoroscopy. Ports ((Polysite 3007 ISP, 7Fr, internal volume 0,35 ml, Perouse Medical) were previously to the surgery inserted with ultra-sound guidance and fluoroscopy control of the extremity of the tip. The day of the surgery, port was perfused, after a cutaneous antisepsis with alcoholic polyvidone-iodine solutions, with a 19G Huber needle. The patient was monitored with a PICCO2 device. We alternately connected the device to the thermistor of the CVC or to the thermistor of the port.

Intraoperative fluid infusion was based on the local hemodynamic protocol. We administered 5 ml/kg/h of crystalloid. Fluid challenge consisted of 250 ml of colloid when hemodynamic indicators were in favor of hypovolemia (pulse pressure variation (PPV)>15% and variation of CI>10%). Perioperative and anesthetic management were not modified by the study.

Measurement of CI by TPTD was based on the average of 3 injections of cooled (<6°C) boluses of saline (15 ml) [6]. We obtained a couple of measurements of the CI by successively injecting 3 boluses into the CVC (CI_CVC_) and 3 boluses into the port (CI_PORT_). The six saline boluses were administered within a maximum of 10 minutes and were randomly performed, as soon as the blood temperature had returned to its baseline value, as indicated by the device.

After the 3 injections of cooled saline into each venous access, the following data were collected: Cardiac Index (CI_PORT_ and CI_CVC_), global end-diastolic volume index (GEDVi_CVC_ and GEDVi_PORT_), extravascular lung water index (EVLWi_CVC_ and EVLWi_PORT_) and central temperature variation (ΔT_CVC_ and ΔT_PORT_). Hemodynamic measurements were performed before and after fluid challenge and when the clinician estimated they were necessary. All data were collected manually on a data entry form during the surgery. There were aggregated in an anonymous database using an Excel spreadsheet (Microsoft, Richmand USA). No deviation from the trial study protocol was noticed.

The primary endpoint of the study was to assess the agreement between the measurements of the CI by TPTD via a port versus a classic CVC.

### Statistical Methods

Biases and limits of agreement were assessed according to the Bland-Altman method for repeated measurements [7]. The linear correlation between the measurements of the CI_CVC_ and the CI_PORT_ was calculated. The percentage error was the relationship between two standard deviations of the difference in the mean of both devices. We determined the 30% threshold, as previously described by Critchley [8]. Trending ability was assessed by plotting ΔCI_CVC_ versus ΔCI_PORT_ on a four quadrant plot. Then concordance in the direction of change between consecutive paired readings from the CVC and the port was scored as a percentage of the total number of paired readings. An exclusion zone of 0.5 L/min/m^2^ was used and a concordance rate of >90% was considered for a reliable trending ability [9]. Considering there was no formula for the calculation of the number of patients to realize a Bland and Altman comparison method, so the choice of the number of patients necessary was based on the pre-existing literature. Previous analogous studies with good methodological quality included at least 60 measurements with maximum of 3 measurements per patients [9], [10]. The maximum acceptable difference between the 2 techniques of TPTD was 20%. At least twenty patients were regarded as necessary.

Results are presented as means ± the standard deviation or number (%). A p value of <0.05 was considered significant. A Bonferroni correction for numerous tests was used. The statistical analysis was performed using Graphpad Prism, version 5.0 (San Diego, CA, USA). Raw data are available on Labarchive (DOI 10.6070/H4154F1T).

## Results

**Figure 1:**
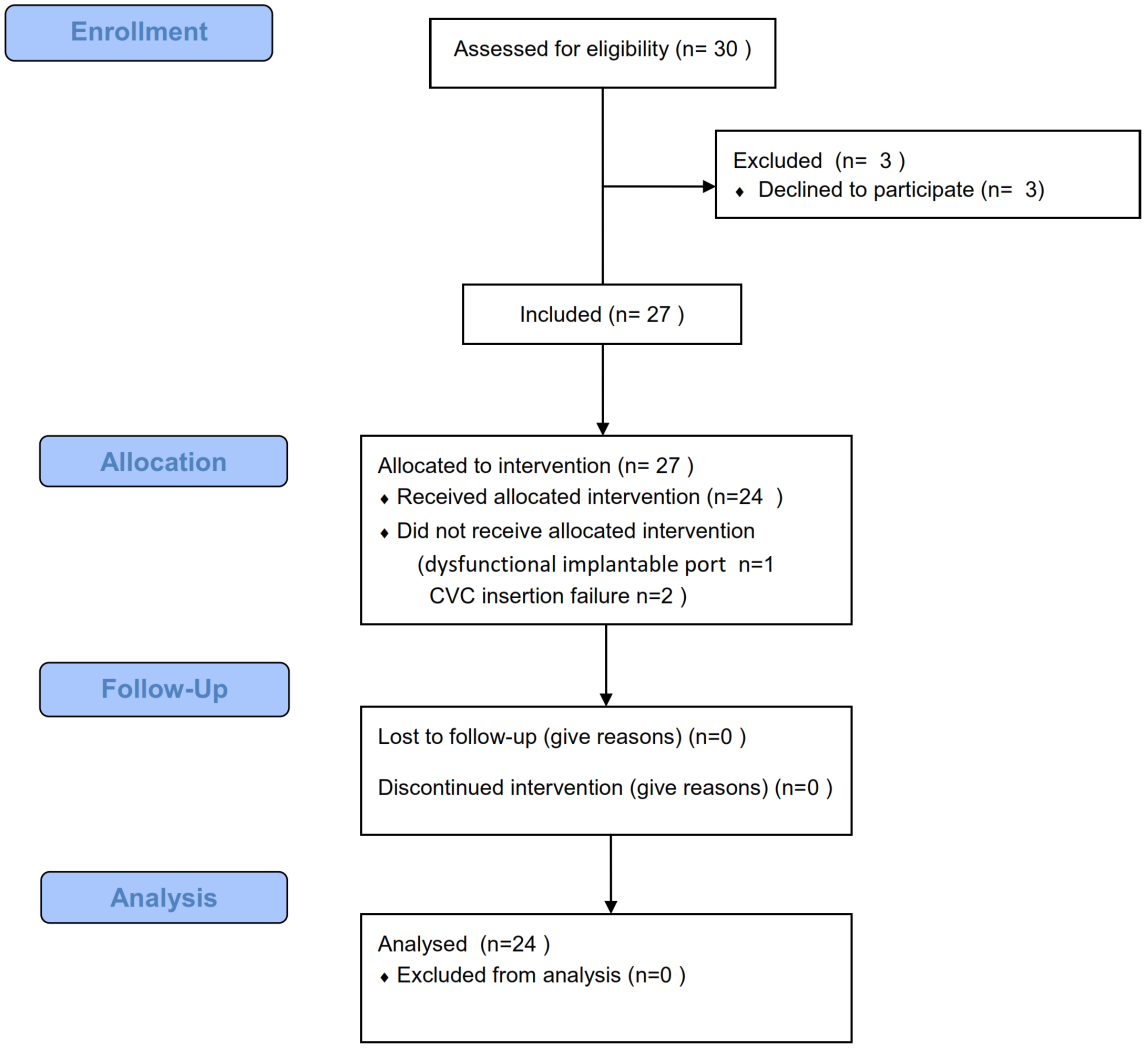
Patients' flow diagram of the study.

The patients' flow diagram is shown in Figure 1.

Twenty-seven women were included in the study and 23 were analyzed. The mean age of the patients was 56±13.58 years. The body mass index was 23.9±3.29 kg/m^2^. One patient was excluded because of a dysfunctional implantable port. Two patients were excluded because we failed to insert the CVC. No port infection was observed during the 30 days following surgery.

The CI was measured before and after sixty fluid challenges and we successfully obtained 123 couples of measurements of the CI. Only one fluid challenge was performed in 3 patients. Table 1 shows the results before and after fluid challenge. CI_CVC_ and CI_PORT_ were similar before and after fluid challenge. CI was significantly increased by fluid challenge, whatever the catheter used (p<0.001). The ΔT_PORT_ was significantly lower than the ΔT_CVC_ before and after fluid challenge (p<0.01). ΔT was never below 0.2°. The EVLWi_PORT_ was higher than the EVLWi_CVC_ (p = 0.01).

**Table 1:** Hemodynamic parameters measured before and after fluid challenge.

	Before fluid challenge		After fluid challenge	
	CVC	Port	CVC	Port
CI (l/min/m^2^)	3.10±0.64	3.28±0.71	3.53±0.61^a^	3.63±0.73^c^
GEDV (ml)	607±92	629±131	646±109	669±120
EVLW	8.09±1.25	8.76±1.7^a^	8.45±1.59	9.17±1.67^b^
ΔT (°C)	0.36±0.06	0.33±0.06^a^	0.34±0.06	0.32±0.05^b^

CI: cardiac index; GEDV: global end diastolic volume; EVLW: extra vascular lung water; ΔT: delta temperature.

a : p<0.01 vs. CVC before fluid challenge.

b : p<0.01 vs. CVC after fluid challenge.

c : p<0.001 vs. Port before fluid challenge.

**Figure 2:**
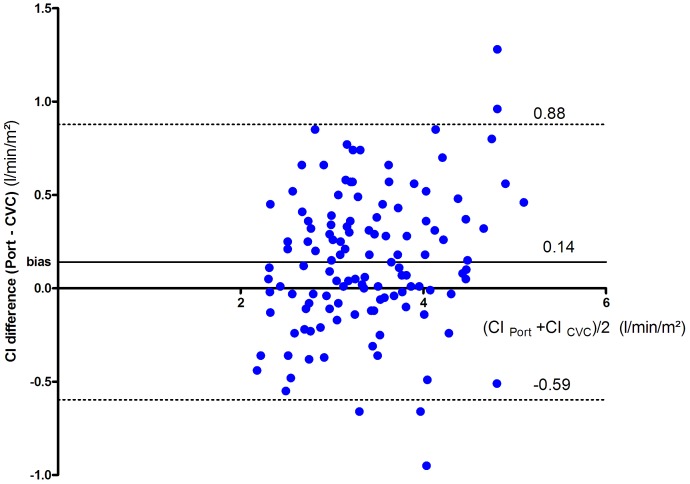
Bland-Altman plot for the Cardiac Index, with a correction for repeated measurements for the calculation of the agreement limits (dashed lines).

The correlation coefficient for the measurement of the CI was 0.87 (95% confidence interval 0.81–0.9). The bias was 0.14 L/min/m^2^ (limit of agreement [−0.59–0.88]) (Figure 2). The percentage error was 22%.

**Figure 3:**
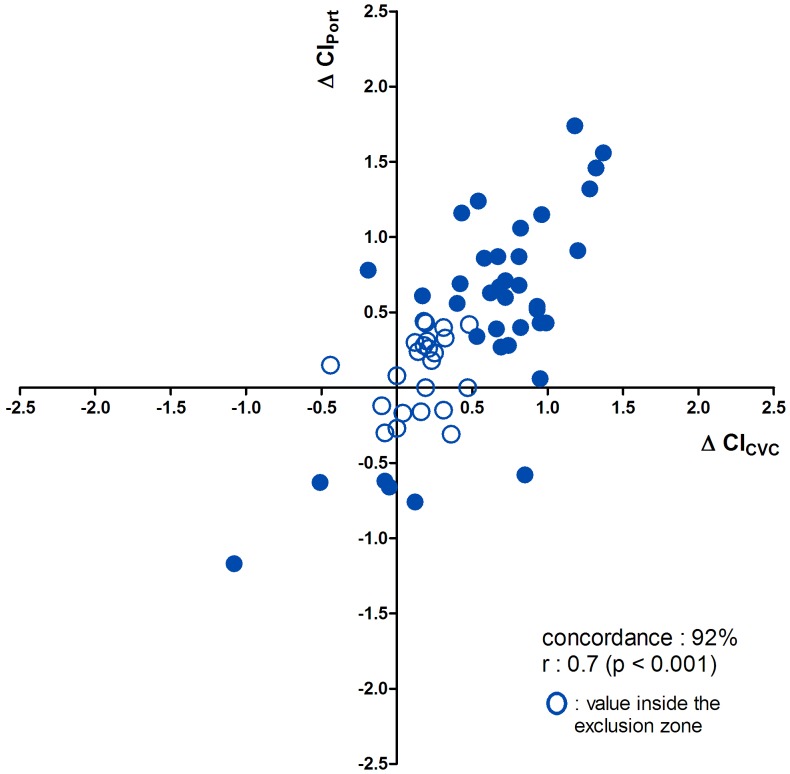
Changes in CIPort readings in comparison with CICVC. Values inside the exclusion zone of 0.5 L/min/m^2^ are grey.

Four quadrant plots are shown in Figure 3. The correlation coefficient was 0.71 and the concordance was 85% without an exclusion zone. The correlation coefficient was 0.7 and the concordance was 92% with an exclusion zone of 0.5 L/min/m^2^.

## Discussion

We showed that measurement of the CI with TPTD via a port was possible and reliable during high-risk oncologic surgery. The concordance of the values is greater than 90% with a bias of 0.14 l/min/m^2^ and the percentage error was 22%.

The ΔT_PORT_ was significantly lower than the ΔT_CVC_ but was never lower than 0.2°C. The ΔT depends on the speed of the injection. This difference shows that the speed of the injection via the port or the CVC is not the same. The volume in the port was less than 0.35 ml (manufacturer data) which cannot totally explain the difference in the ΔT. This difference may be explained by the small internal diameter of the needle connected to the port which could affect the output of the infusion. Nevertheless, it is possible to obtain an output of 5 ml/s with a high viscosity product like a radiological contrast agent [11]. The duration of each cooled saline injection was below 7 s which was according to the PICCO2 manufacturer's recommendations. Consequently, the difference in ΔT observed is not clinically relevant and is probably not an obstacle to using the port to measure IC with TPTD.

The concordance and percentage error values remain outside 90% and 30% benchmarks for acceptable agreement and trending. Measurement of the CI_PORT_ is achievable and similar to that obtained by the CVC.

Measurement of the CI_PORT_ could be used for intraoperative monitoring of high-risk oncologic surgery. Most patients are already fitted with a port which is inserted via the right jugular or subclavian vein. Recently, authors reported difficulties in inserting the CVC via the left jugular vein [12]. We were confronted with the same problem and had to exclude 2 patients because we failed to insert the CVC on the left side even though the clinicians were experienced and used ultra-sound guidance. Measurement via the CI_PORT_ avoids the risks of complications due to the placement of a new CVC as pneumothorax, arterial puncture, catheter malposition, hemothorax, vessel stenosis or thrombosis, local hematoma and local or general infections [13]. The main limit of the technique is the absence of a CVC for eventual catecholamine administration because port comports only one lumen so that if catecholamine are administered by the port, TPTD becomes impossible by the port (major risk of variation of the administration of the drug). We do not recommend the exclusive use of the port for TPTD. We identified local intermediate-risk and high-risk patients. For intermediate-risk cases as oncologic important surgery for patients without major comorbidities (ovarian cancer cytoreductive surgery, right hepatectomy, hyperthermic intraperitoneal chemotherapy for colic carcinomatosis, pancreatic surgery, oesophagectomy…), we propose using the port to measure the CI and to administer catecholamines via a peripheral venous access if necessary. For high-risk cases as very specific oncologic surgery (cytoreduction of major abdominal mesothelioma, abdominal surgery programmed longer than 10 hours, major abdominal sarcoma greater than 10 kg…) or surgery for patients with major comorbidities, we propose inserting a femoral venous and arterial catheter. Placement of the femoral catheters is safer and easier [14], [15]. During the study, no patients required catecholamines. Measurement of the CI_PORT_ allowed us not to insert an additional CVC for intraoperative monitoring.

Our study had some limitations. Femoral arterial catheter could be more invasive than radial arterial catheter. Femoral arterial catheter does not increase the risks of complications (hematoma, member ischemia) [16]. Infectious risks are not more important with femoral access [17]. In our study, we did not observe any catheter infections during the month following surgery. Risks of implantable port infections increase with the number of times the device is used [18]. We did not observe an increase in catheter-related infections probably because nurses and doctors are used to managing patients with ports and respect the local protocol of management of the venous catheter. Implementation of a local protocol of management of the central venous catheter in intensive care allowed to decrease infection [19]. Anesthetist team is used to manage port intraoperatively: port is strictly reserved for intraoperative chemotherapy and strict hygienic measures are respected. We applied the same protocol for TPTD: no injection of drug by the venous catheter except saline boluses for TPTD. Our study was not designed to show an increase in the catheter-related infections.

## Conclusions

Intraoperative CI can be measured with an implantable port. Insertion of an additional CVC is not necessary when patients have a port for oncologic treatment. Other studies are warranted to confirm the safety of the technique.
